# Regulatory, Legal, and Market Aspects of Smart Wearables for Cardiac Monitoring

**DOI:** 10.3390/s21144937

**Published:** 2021-07-20

**Authors:** Jan Benedikt Brönneke, Jennifer Müller, Konstantinos Mouratis, Julia Hagen, Ariel Dora Stern

**Affiliations:** 1 Health Innovation Hub, Torstr. 223, 10785 Berlin, Germany; julia.hagen@hih-2025.de (J.H.); ariel.stern@hih-2025.de (A.D.S.); 2 Helios Health Institute, Helios Health, Friedrichstraße 136, 10117 Berlin, Germany; jennifer.mueller@helios-health.com; 3 Herzzentrum Leipzig, Universitätsklinik für Kardiologie, Strümpellstraße 39, 04289 Leipzig, Germany; konstantinos.mouratis@leipzig-heart.de; 4 Leipzig Heart Institute, Russenstraße 69a, 04289 Leipzig, Germany; 5 Harvard Business School, Harvard University, Morgan Hall 433, Soldiers Field Road, Boston, MA 02163, USA; 6 Hasso-Plattner-Institute, Prof.-Dr.-Helmert-Straße 2-3, 14482 Potsdam, Germany

**Keywords:** medical devices, regulation, market access, smart wearables

## Abstract

In the area of cardiac monitoring, the use of digitally driven technologies is on the rise. While the development of medical products is advancing rapidly, allowing for new use-cases in cardiac monitoring and other areas, regulatory and legal requirements that govern market access are often evolving slowly, sometimes creating market barriers. This article gives a brief overview of the existing clinical studies regarding the use of smart wearables in cardiac monitoring and provides insight into the main regulatory and legal aspects that need to be considered when such products are intended to be used in a health care setting. Based on this brief overview, the article elaborates on the specific requirements in the main areas of authorization/certification and reimbursement/compensation, as well as data protection and data security. Three case studies are presented as examples of specific market access procedures: the USA, Germany, and Belgium. This article concludes that, despite the differences in specific requirements, market access pathways in most countries are characterized by a number of similarities, which should be considered early on in product development. The article also elaborates on how regulatory and legal requirements are currently being adapted for digitally driven wearables and proposes an ongoing evolution of these requirements to facilitate market access for beneficial medical technology in the future.

## 1. Introduction

This article provides an overview of the most relevant regulatory, legal, and market aspects impacting smart wearables for cardiac monitoring. Given the wide variety of regional and country-specific regulatory, legal, and market access frameworks that exist worldwide, this article will focus on the European and U.S. contexts. However, the issues discussed, and their corresponding regulatory responses, are transferable to most countries, especially those with a regulated health care delivery system and product market. 

The most important regulatory aspect for wearables used for cardiovascular diseases is their market access through certification or authorisation. The most prominent of these processes are European CE-marking via a conformity assessment procedure and clearance or approval by the U.S. Food and Drug Administration (FDA). For most devices and medical equipment used in health care, this is the first step in market access. 

One of the most important questions for device manufacturers is: who is going to pay, and in which way, for the acquisition and ongoing use of a device? The answer is highly dependent on the respective health care system, which, in turn, will often set prerequisites for the use of the devices in practice (that is, beyond the local regulatory requirements). 

Beyond the two market access questions of (1) authorisation/certification and (2) reimbursement/compensation, other legal questions arise for manufacturers marketing their smart wearables. Most prominently, these concern the privacy and security of the processed data, which are indirect requirements of the market access process. Once marketed, other legal requirements arise regarding the application of the device, such as liability for malfunction, the professional regulations of medical users, and the data processed. The subset of legal requirements that should be considered during the market access process (but are not established requirements within this process) will not be covered by this article.

**Figure 1:**
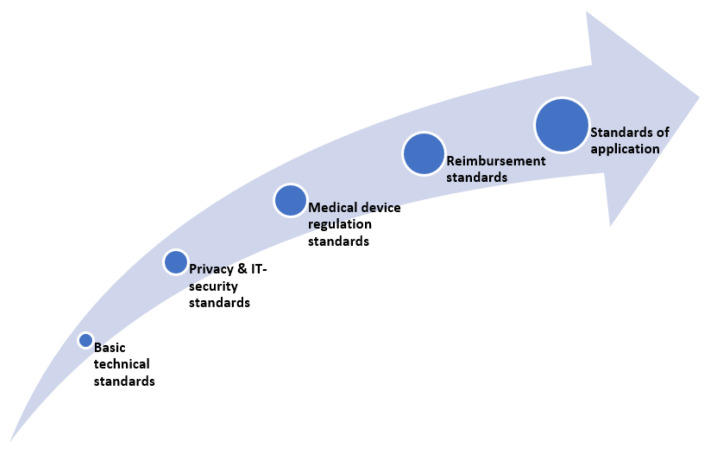
Regulatory and legal aspects of market access of smart wearables.

All of these considerations and their respective procedures can be understood as part of the market access pathway (see Figure 1) and are closely related and dependent on one another. As such, their consideration at an early stage in the development of smart wearables is strongly recommended.

## 2. Background and Definitions

The use of digital tools in health care is now ubiquitous and raises several questions around practical topics, including technical specificities, privacy and data security, assessment of clinical safety and efficiency, patient benefits in the realm of regulation, legal frameworks, and market access for such tools. Many digital tools use sensors to collect the data needed for their functionality. Existing digital tools and their associated sensors now address the broadest imaginable range of health-related issues and medical conditions and, therefore, come in a wide variety of shapes and uses.

**Figure 2:**
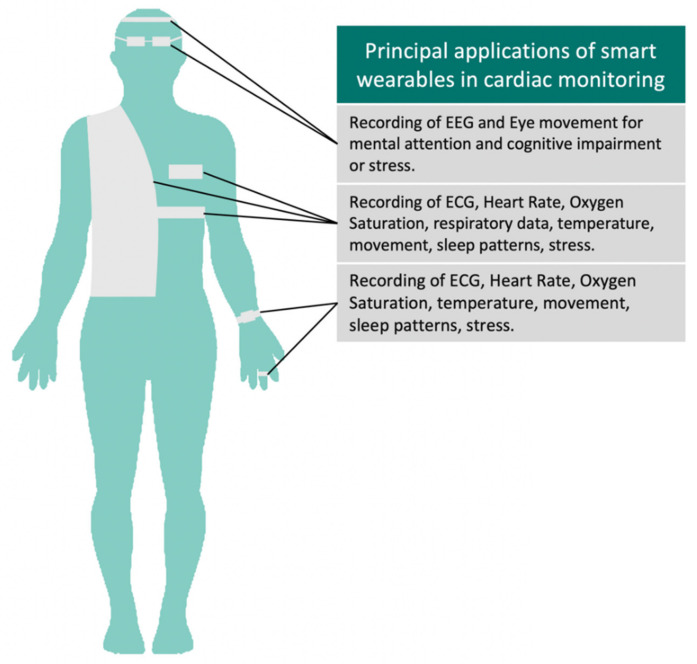
Applications of smart wearables in cardiac monitoring. Different devices can be adapted to textile or everyday items such as watches, glasses, or rings to fit the patient’s style and comfort. These can capture a variety of important physiological and behavioral parameters.

In this article, we discuss the regulatory, legal, and market aspects of the internet- or network-connected devices used for remote the biometric monitoring of patients [1] suffering from cardiovascular diseases. Goldsack et al. (2020) defines Biometric Monitoring Technologies (BioMeTs) as follows: “BioMeTs are connected digital medicine products that process data captured by mobile sensors, using algorithms to generate measures of behavioural and/or physiological function” [2]. Our main focus is on a subset of these devices, generally acknowledged as “smart wearables”. Such products can be typically be worn epidermally or be placed in body cavities, such as the ear or mouth [3]. Such devices can be either consumer-grade or medically-certified or authorized (see Table 1), thus including gadgets such as watches, glasses, clothing, etc. that incorporate sensors to capture kinetic, behavioural, or physiological data [4] with the use of appropriate software (see Figure 2).

### 2.1. Scenarios of Use

We provide more detail on the possible use scenarios by illustrating the current market trends. This serves as both the validation of existing products, as well as inspiration for future researchers, innovators, and scientists who wish to develop solutions for mobile health treatment, monitoring, and management of patients.

Given this aim, rather than identifying use cases from the scope of a given device, we consider the application of these solutions in a clinical setting. This is particularly important, given that the wearable-based mobile health solutions adoption rate in the clinical setting is still falling short of expected results, despite their ongoing development [15]. For this exercise, we performed a text search on clinicaltrials.gov, with the goal of identifying clinical trials that represent the ways in which health care providers (HCPs) may adopt such technology in the clinical (research) setting. 

### 2.2. Methods

We used the search function of clinicaltrials.gov to identify studies with a status of Active, Enrolling, Recruiting, or Completed, including the term “wearable” and filtered for at least one condition or disease listed included the category “Cardiovascular Disease”. No further criteria limiting patient age, sex, or trial location and type were applied. As of March 2021, our search provided an output of 203 clinical trials from the years 2011 to 2021, which were downloaded as a structured text file for manual quality checks and filtering.

### 2.3. Results

**Figure 3:**
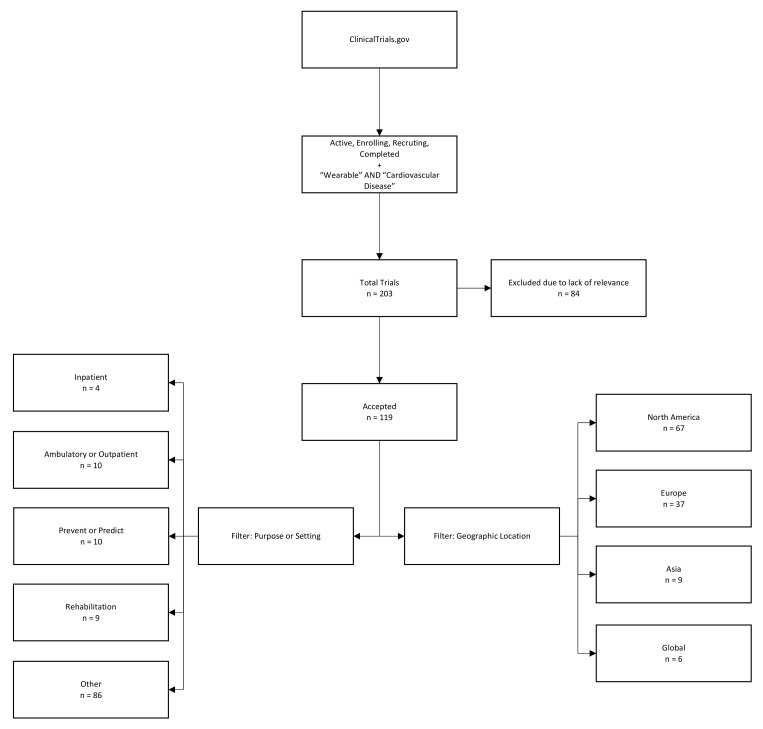
Methodology of identification of clinical trials related to cardiac monitoring with the use of wearables via clinicaltrials.gov and accompanying data.

Of the 203 trials we identified as being representative candidates for the use of smart, connected wearables for cardiac monitoring, 84 were excluded due to lack of relevance to our setting, while the remaining 119 were checked using text filters to identify patterns of use and setting. The geographic location was taken from trial information and clustered into the following regions: North America, Europe, Asia, and the rest of the global market (Figure 3).

### 2.4. Findings and Discussion

The results presented in Figure 3 suggest a series of trends regarding wearables in the clinical setting. Firstly, only a small share (four of 119 cases (or roughly 3.4%)) appeared to be targeted at inpatient treatment. Instead, in the cardiac setting, most wearables seem to be used for outpatient monitoring or in other settings; for example, as a method to prevent patients from experiencing future serious events, either by motivating change of behaviour or enabling prediction, a second important, but unsurprising finding, is the distribution of the markets in which medical research on wearables is conducted (and, thus, usually indicating the markets that such products are intended for); most clinical trials in recent years have been based in North America (56.30%), followed by Europe (31.09%), and Asia (7.56%). 

Notably, this does not indicate that market access in North America is easier (nor anything about the degree of competition), as these topics are beyond the scope of this simple analysis. Nevertheless, these preliminary descriptors of the clinical trial landscape hint at trends in the types of studies being performed and where they are taking place. Future researchers may wish to do a more in-depth analysis of the break-down of the study sponsors, as well as how such tools are used in clinical trials. For example, Marra et al. (2020) defines four possible use scenarios for connected digital products (broadly) in clinical trials, noting the distinctions between digital tools that are themselves the intervention and those that are used to capture endpoint data for another intervention (e.g., an exercise program or pharmaceutical product), among other things [16]. In their study, Marra et al. documented a 34% cumulative annual growth rate in the use of connected digital products in clinical trials over the past two decades, suggesting that this will be a growing area of application for such tools (notably, the Marra et al. study is based on data from before the COVID-19 pandemic and, therefore, does not yet capture any additional increases in the use of connected digital products in clinical trials that may have emerged since the pandemic’s onset.).

In particular, further research into the possible scenarios of use for connected wearables for cardiac monitoring may serve to either support current trends, such as the use of photoplethysmography for the detection of arrhythmias [17], or give way to new paths, such as ambulatory telemedicine, a setting which became increasingly necessary during the COVID-19 pandemic [18]. Such approaches can also lead to the secondary outcome of reducing health care costs, provided that approval and reimbursement proceeds as expected [19]. 

## 3. Regulatory Aspects of Smart Wearables

Smart wearables may qualify as medical devices, according to the definitions of the applicable regulations for Europe, North America, China, and other countries. In this section we will focus on the new European Medical Device Regulation 2017/745 (MDR), in which a product must comply with the MDR if it falls under the following definition:

“*Any instrument, apparatus, appliance, software, implant, reagent, material, or other article intended by the manufacturer to be used, alone or in combination, for human beings for one or more of the following specific medical purposes: Diagnosis, prevention, monitoring, prediction, prognosis, treatment, or alleviation of disease […] and which does not achieve its principal intended action by pharmacological, immunological, or metabolic means, in or on the human body, but which may be assisted in its function by such means […]*”

(MDR, Art. 2(1)) [20]

Notably, the definition of a medical device are not identical between the EU’s MDR and the Medical Device Amendments of the U.S. Food Drug and Cosmetic Act (U.S. FD&C Act) (see Table 2). Additionally, the definition for software is much more complex under the EU-MDR. 

Smart wearables, without a medical use (including those that are lifestyle or fitness products), are out of scope of the regulatory section of this publication. Additionally, analysis regarding the regulatory changes for in vitro diagnostic medical devices also falls out of the scope of this article and is, therefore, not discussed. 

### 3.1. MDR Requirements for Medical Software Developers

Currently, manufacturers who intend to place, or who have already placed, medical devices on the European market face major challenges, due to a changing regulatory landscape. Many of the policies that are novel and are raising questions today were set into motion by the Poly Implant Prothesis (PIP) scandal of 2010 (in which breast implants were produced with cheaper, industrial-grade silicone that was not approved for medical use, leading to severe, adverse events and product recalls for patients based on incident reports; these implants were reported by the European Scientific Committee on Emerging and Newly Identified Health Risks) and the changes continue to this day. 

In general, for the review and approval of medical devices in the European Union (EU), there is no centralized regulatory body, such as the European Medicines Agency (EMA), which regulates drugs, or the U.S. FDA, which regulates foods, medicines, and medical devices in the United States. Instead, under European regulations, a medical device manufacturer must declare conformity to applicable regulations and standards. Additionally, contingent on the medical device’s risk class, a “Notified Body” must be involved in the conformity assessment procedure. The EU defines a Notified Body as “an organisation designated by an EU country to assess the conformity of certain products before being placed on the market” and publishes a list of such Notified Bodies [22].

In 2020, the COVID-19 pandemic highlighted the need for medical innovations ranging from protective equipment to stable health care systems. At the same time, the three-year transition period for the new Medical Device Regulation 2017/745, which was adopted in Europe and entered into force on 25 May 2017, was extended by one year in order to provide the bloc’s health care system and medical device manufacturers with flexibility during the pandemic.

The MDR defines the regulatory requirements for medical devices, accompanying harmonized standards, common specifications, and guidance documents from the Medical Device Coordination Group (MDCG) of the European Commission. These apply for all devices (aspiring to be) legally placed on the market in the European Economic Area (EEA). The transition period of the changeover from the older Medical Device Directive 93/42/EEC (MDD) and Active Implantable Medical Device Directive 90/385/EEC (AIMDD) to the EU MDR 2017/745 ended on 25 May 2021 [23]. At the latest, the new regulation must be implemented in all EU and EEA member states from this date onward. 

While the regulatory system, in general, is not completely novel for manufacturers who previously marketed products of higher risk classes under the previous MDD, the MDR increases requirements (among other things) for medical device traceability, post-market surveillance activities, and medical device software products. The impact of the MDR is particularly acute for software manufacturers. The MDR broadens the definition of a medical device and considers, in greater detail, new technologies such as medical software. Specific rules for the classification of medical device software can be found in the MDR in rule 11 of chapter III of Annex VIII [20].

On a product specific, case-by-case basis, products such as smart wearables must fulfil the requirements of the MDR and must bear a CE mark if the manufacturer claims that their product has a medical purpose and this purpose corresponds to the definition of a medical device, according to the MDR (MDR, Art.2(1)) [20]. Whether a product qualifies as a medical device is determined by its intended purpose.

#### 3.1.1. Key Characteristics of Software Qualification under MDR

The International Medical Device Regulators Forum (IMDRF) defines Software as a Medical Device (SaMD) as “software intended to be used for medical purposes that performs its objectives without being part of a hardware medical device” [24]. Under EU-MDR guidance documents, the definition is broader and states that “medical device software is software that is intended to be used, alone or in combination, for a purpose as specified in the definition of a “medical device” in the MDR or IVDR, regardless of whether the software is independent or driving or influencing the use of a device” [25]. As a result, the EU regulation is applicable to stand-alone software, embedded software, and beyond.

To qualify as medical device software (MDSW), according to the guidance document on the qualification and classification of software by the MDCG, the key questions are whether the software in question performs an action on data that is for a medical purpose beyond archiving, communication, storage, or simple search of data and if the performed action is directed towards, and for the benefit of, an individual patient [25]. When this is the case, a product most likely qualifies as a medical device and is subject to the requirements of the MDR. Rule 11 of the MDR sets specific classification rules for software that is intended for decisions with diagnosis or therapeutic purposes and beyond. The respective MDCG document guides manufacturers in this regard and describes and categorizes the significance of the information provided by the active device (here, software) to the health care decision (patient management), in combination with the health care situation (patient condition) [25].

This new rule implies that many software products with a medical purpose may have to be (re)classified more stringently as class IIa, IIb, or III under the MDR, while under the previous MDD, most standalone software products were classified as (lower risk) class I devices [26]. 

For the manufacturers of smart wearable devices, this means that as soon as a software product falls into risk class IIa, according to the Regulation (at a minimum), its manufacturer must meet more significant regulatory requirements in order to demonstrate general safety and performance requirements (GSPRs) before legally placing its product on the European market. 

Regarding cybersecurity issues, potential risks of cyber threats or unauthorised access to data should also be considered and minimized by manufacturers across the total product life cycle. As Gordon and Stern (2019) noted, networked devices are particularly susceptible to “major cybersecurity risks by creating new attack surfaces and vulnerabilities that are not present in isolated devices” while an additional and growing cybersecurity risk is the reliance of many products on third-party, off-the-shelf components [27]. Future initiatives to ensure software quality and reduce vulnerabilities may include such approaches as the publication of a “software bill of materials” (SBOM). As Carmody et al. (2021) explained, “the risk of including third-party software components in health care technologies can be managed, in part, by leveraging a software bill of materials (SBOM)…SBOMs provide a transparency mechanism for securing software product supply chains by enabling faster identification and remediation of vulnerabilities, towards the goal of reducing the feasibility of attacks” [28].

#### 3.1.2. Clinical Evaluation as Part of the GSPRs

To meet the relevant GSPRs, medical device manufacturers must also evaluate the undesirable side-effects and acceptability of the benefit-risk-ratio, based on clinical data according to a clinical evaluation planned and documented by the manufacturer (MDR, Art. 6) [20]. In the clinical evaluation, manufacturers of medical device software need to demonstrate the scientific, technical, and clinical validity of the software [29]. New data on a device from pre-market testing or from post-marketing activities, as well as considerations for new or changed intended purposes, require an updated clinical evaluation report and may indicate the necessity of clinical investigations (MDR, Art. 61) [20]. The manufacturer may have to perform (further) clinical investigations, depending on the clinical claims made by the manufacturer, the risk class of the device, the results of the risk analysis, and the clinical evaluation. The clinical evaluation is best understood as a fully comprehensive process over the whole product life cycle, with the goal of ensuring ongoing safety and performance, rather than as a standalone, point-in-time report. New approaches using real-world data (RWD) may be an opportunity to generate the needed clinical evidence, as provided for in Germany’s Fast-Track (see Section 6.2, Case Study 2), which facilitates the use of RWD. 

#### 3.1.3. Medical Device Software Life Cycle

Developers and manufacturers must ensure that their software development processes follow the principles of a software lifecycle within a quality management system to comply with the regulations. In this regard, manufacturers should be familiar with existing standards (e.g., the ISO IEC 62304:2006 for medical device software standardizing life cycle processes, which consists of software development, validation and verification, software maintenance, problem resolution, risk management, and configuration management) [30]. 

### 3.2. Key Take-Aways for MDSW Manufactures under the EU-MDR

The involvement of a Notified Body for the conformity assessment procedure under the MDR will become mandatory for most medical device software manufacturers to be allowed to affix a CE mark to their product(s). On the manufacturer side, this involvement can lead to protracted periods of time until a medical device can be placed on the market. Developers and manufacturers of smart wearables that include software with an intended medical purpose should plan to begin addressing and planning for the regulatory requirements of the MDR at a very early stage. This will allow time for generation of the required evidence and documentation throughout the product life cycle and within the framework of an appropriate quality management system.

Innovative and technology-driven products with a short product lifecycle (including, but not limited to, smart wearables) may be meaningfully slowed down, therefore reducing incentives for innovation and commercialization in the European health care sector. Against this backdrop, some countries, such as Germany, are taking steps to create new options and pathways for evidence generation for the digital medical devices of lower risk classes, in order to collect clinical data in a way that supports innovation and entrepreneurship. Germany’s DiGA Fast-Track is described in more detail in the country case studies below. For manufactures of higher risk products, the MDR may make it more difficult to be able to secure competitive advantages and alternative pathways for evidence generation that do not yet exist.

### 3.3. Regulatory Pathways in the United States: Clearance and Approval

Each manufacturer chooses target markets for their products; while some are focused on certain markets initially, manufacturers typically aim to expand into new markets as companies grow. In addition to the European CE region, the North American market can be of particular interest for medical device manufacturers. The United States, in particular, is the world’s largest medical device market and is typically quite attractive for device manufacturers worldwide. 

In the United States, medical devices are regulated by the FDA, as laid out in the 1976 Medical Device Amendments to the Federal Food Drug and Cosmetic Act (FD&C Act) [21]. All medical device companies and their products must be registered with the FDA, a requirement to market and sell medical devices of all risk classes in the United States. Medical devices can be “FDA Registered”, “FDA Cleared”, or “FDA Approved” [31], depending on their associated risk class (class I, II, or III, respectively) and additional requirements. It is important to note that the classification rules for US device risk classes are not the same as those established in other regulations, such as the EU-MDR. 

The “FDA Registered” label on a device typically indicates low risk and the designation that the FDA is aware of the manufacturer and their devices. Class I devices that present a low risk (such as surgical gloves) are subject to general controls only (e.g., compliance with good manufacturing practices) and most are exempt from premarket notification requirements. Medical devices that are not generic, or otherwise exempt, must go through a premarket regulatory process.

Class II (typically moderate risk) devices (such as blood pressure cuffs) that are not exempt require that the FDA “clears” a product for marketing. The premarket clearance process is often called the 510(k) process, after the section of the FD&C Act establishing this pathway [32]. This is typically performed through the demonstration of the “substantial equivalence” to one or more already legally marketed predicate device(s) that do not require (the more stringent) premarket approval and are labelled as “FDA Cleared” [31]. Most of the devices listed in Table 1 were cleared through the FDA’s 510(k) process and have followed this regulatory pathway. 

The final category is premarket approval (PMA), often referred to as the “PMA process” [33]. The Omron Heart Failure Watch is an example of an FDA-approved device (see, Table 1). Here, according to the regulations governing devices of the highest-risk (class III, which are often implantable and/or life-sustaining), manufacturers must go through the PMA process before they can legally market a device. To receive premarket approval, manufacturers must demonstrate with sufficient, valid scientific evidence that there is a reasonable assurance that the device in question is safe and effective for the intended use set by the manufacturer. This is typically done through clinical trials to demonstrate a positive risk-benefit profile for the patient, in addition to bench-top testing and other device-appropriate controls (e.g., biocompatibility testing of the materials that come, directly or indirectly, into contact with patients or users). 

Clinical trials of high-risk devices are often time-consuming and costly; as such, devices that go through the PMA process typically spend longer in both regulatory approval and in pre-approval activities, including (pivotal) clinical trials. Notably, the most novel medical devices have been shown to experience longer periods of FDA review. Indeed, Stern (2017) showed that among PMA-track devices, so-called “pioneer entrants” (first-in-class devices) “spend 34% (7.2 months) longer than follow-on entrants in regulatory approval” [34].

## 4. Subsequent Market Access Considerations Regarding Remuneration 

Depending on their intended scenarios of use, smart wearables with a medical purpose often must fulfil additional requirements to be remunerated (reimbursed or compensated) within country-specific, legally framed (public) health care systems. While a regulatory authorisation/certification is a *conditio sine qua non* for market access of such sensors, it does not necessarily imply a direct claim to remuneration in such systems. The specific requirements are usually dependent on:

- the specific health technology in question (drug, medical device, hardware, and software),
- the specific structure of the health care system in question and its financing mechanisms (e.g., a mix of public and private insurers, as in the United States, statutory/public insurers, as in Germany and France, or state-driven and tax-funded insurers, as in the United Kingdom and Nordic countries),
- the actors responsible for assessing the technology (e.g., insurers and regulatory authorities) and
- the specific scenarios and medical contexts of use (e.g., in-patient vs. out-patient settings, prevention, diagnosis, treatment, and rehabilitation).

Despite differences in the specific requirements and procedures for reimbursement or compensation for smart wearables used for medical purposes, most are based on the same assumptions and have common foundations. First and foremost, financial constraints in most health care systems require a (centralized) remuneration decision based on an assessment of the benefits of health technologies (Health Technology Assessment, HTA). While HTA should consider several different aspects of the technology in question, such as its technical description, its safety (both being focused on in medical device regulation), ethical, legal, and social implications, the assessment of the clinical effectiveness or clinical benefits lie at the very core of each HTA, often accompanied by an analysis of a product’s economic effectiveness. Although aspects considered in the remuneration decision, such as proof of clinical effectiveness, are—to a certain extent—part of the authorisation/certification process, the requirements for reimbursement can differ from the regulatory requirements for legal marketing and are sometimes significantly stricter than the latter.

### 4.1. Clinical Efficacy and Effectiveness

While clinical effectiveness refers to the benefit of patients under the circumstances of actual health care provision, clinical efficacy refers to the effectiveness within the conditions of a clinical trial or laboratory test. The term “clinical effectiveness” is mostly used synonymously with the term “clinical benefit”, but is clearly distinct from the term “clinical efficacy”. The clinical effectiveness of a medical device is defined as its capability to induce the intended positive clinical effects (assuming they outweigh potential negative side effects). Although clinical effectiveness is arguably the more important indicator of how well a product works in practice [35], for methodological reasons, remuneration decisions are largely based on evidence from clinical trials and, therefore, are based on the clinical efficacy of the technology in question. 

Clinical efficacy or effectiveness of a technology can be demonstrated based on clinical studies that investigate a technology’s effect on relevant clinical endpoints in the areas of mortality, morbidity, adverse effects, and health-related quality of life (or, in the case of diagnostic tools, the technology’s sensitivity and specificity). Studies are usually ranked according to the fundamental principles of Evidence-Based Medicine (EBM) [36]. A core principle of EBM is the ranking of such studies, according to the internal validity of study designs, meaning that randomised controlled trials (RCT) are ranked higher, in terms of their informational value, than non-randomised trials, retrospective observational studies, case studies, or, at the bottom of the hierarchy, expert opinions [37]. 

In most cases in the EU, at least one RCT (or even a systematic review of several RCTs) is needed to sufficiently prove a clinical benefit for a positive remuneration decision for high(er) risk devices. In some countries, moreover, the competent bodies request not only proof of clinical efficacy or effectiveness, but also a comparison of the technology’s efficacy with the efficacy of alternative, already existing diagnostic or treatment options. Furthermore, the assessment of clinical benefits might not be limited to the effects of the device itself, but might include the health care delivery method or context in which a device is used. For example, the assessment for a smart wearable for cardiac home-monitoring should arguably encompass the clinical benefit of home-monitoring for the specific disease, rather than the broad use of the device itself. 

### 4.2. Cost-Effectiveness

A technology’s cost-effectiveness is mostly understood as its efficiency (i.e., its ratio of clinical benefit, for example, measured in quality adjusted life years (QALYs) or other appropriated endpoints) to its cost [38]. Although, the assessed cost-effectiveness of health technologies is crucial for remuneration decisions, in light of the financial constraints of health care systems, it can only be evaluated as a second step after a clinical benefit has been demonstrated. As noted above, cost-effectiveness is often assessed in relation to existing diagnostic or therapeutic options (to the extent that comparator technologies exist). The assessment of cost-effectiveness faces several difficult appraisal decisions, particularly concerning the (monetary) value of a clinical benefit. 

Cost-effectiveness is usually assessed on the basis of health economic evaluations, using statistical models and theoretical decision analysis [39]. Due to the different outcome measures used for different diseases, it is sometimes difficult to compare results *across* different conditions. As such, the uses of economic evaluation methods are often disease- and decision-specific, which leads to a diversified landscape of models (e.g., screening for cardiovascular diseases [40], chronic obstructive pulmonary diseases [41], or for risk prediction modelling [42]).

## 5. Privacy and Data Security

Beyond market access (i.e., aspects of medical device regulation and remuneration decisions within a health care system), privacy (or data protection) and data security regulations, as well as best practices are paramount for smart wearables intended to be used in a medical context. In Europe, the United States, and many other regions, specific legal regimes protect the privacy of citizens and include sensitive data, such as health care data.

### 5.1. Regulations in Europe

In Europe, the European General Data Protection Regulation ((EU) 2016/679, GDPR) is the general and directly binding framework for processing (wholly or partly by automated means) the personal data of any person within the EU and by any processor in the EU (Art. 3 GDPR) [43]. Personal data are defined as “any information relating to an identified or identifiable natural person” (Art. 4, Nr. 1 GDPR). The GDPR applies to a wide variety of software-driven devices [44], including smart wearables [45].

The GDPR grants the data subject extensive rights to get information about, change, or delete processed data, as well as to restrict its processing to specific purposes (Artt. 12–23 GDPR). The controllers and processors of data are correspondingly obliged to inform the data subjects fully, take precautions to protect personal data by design and default with technical and organisational measures, extensively record processing activities, and to ensure data security (Artt. 24–43 GDPR). For example, using encryption methods, which possibly lead to data not being considered personal data anymore, with the consequence of the GDPR not being applied to them in parts [46]. 

One of the more controversial rules of GDPR states that controllers and processors must ensure that data are only transferred to third countries or international organisations if these states or organisations comply with the GDPR or ensure an equally high standard of data protection (Artt. 44–50 GDPR). In its judgement “Schrems II” [47], the European Court of Justice determined that the United States does not fulfil the high standards of GDPR; this ruling affects the use of, for example, U.S.-based cloud data storage services, by requiring extensive contract clauses regarding the processing of personal data [48]. Without such clauses, even the simple storage of health care data of European users (for example, of smart wearables for cardiac monitoring) on U.S.-based clouds (such as Azure, AWS, or the like) is prohibited. This decision will apply until a time when the EU commission passes a new “adequacy decision” regarding data transfers to the United States. 

Non-compliance with the GDPR can be sanctioned by competent authorities with fines up to 20 million Euros or four percent of the total worldwide annual turnover of the preceding financial year of the company in question, whichever is higher (Art. 83 GDPR). Obligations and technical and organisational measures must be appropriate, with regard to the assessed risks, which are generally higher when processing “special categories of data”, such as health data (Art. 9). Overall, the GDPR allows the processing of personal data only when the data subject gave informed consent or if a specific legal norm allows the processing without consent. The GDPR furthermore allows for further national laws for the specific processing of special categories of personal data, meaning that producers of smart wearables must comply with these national rules as well, depending on the target market. 

Lastly, it is worth noting that GDPR-compliance is not only mandatory for CE-marked medical devices, but also for all products that allow the processing of personal data (especially health data), even if they are so-called lifestyle products serving non-medical purposes. The GDPR requirements for smart wearables for cardiac monitoring are accordingly high and should be considered by manufacturers and developers at an early stage of product development in order to successfully reduce privacy concerns down the road vis-à-vis patients, health insurers, and clinicians [49].

### 5.2. Regulations in the USA

While not as strict as the European GDPR, the legal data-protection regime in the United States also includes specific rules regarding health care data, in particular, the Health Insurance Portability and Accountability Act of 1996 ((HIPAA). Sec. 264 a) of the act commissions the Secretary of Health and Human Services (HHS) to define recommendations for “standards with respect to the privacy of individually identifiable health information”.

Subsequently, the HHS published the “Standards for Privacy of Individually Identifiable Health Information” (HIPAA Privacy Rule), which establishes national standards for the protection of identifiable health information, as well as the “Security Standards for the Protection of Electronic Protected Health Information” (HIPAA Security Rule, both known under the term “administrative simplification provisions”), which contain a set of security standards for protecting identifiable health information that is held or transferred in electronic form. 

The two rules contain specific measures to be taken by HIPAA “covered entities”, which are defined as “(1) health plans, (2) health care clearinghouses, and (3) health care providers who electronically transmit any health information in connection with transactions for which HHS has adopted standards” [50]. Manufacturers of wearables that contract with any of these parties for device use, therefore, fall under HIPAA rules [51]. 

Much like the GDPR, the privacy rule distinguishes between the use of protected health information (PHI), based on a permission by the rule itself and use based on the (written) consent of the subject of the information. However, unlike the GDPR, HIPAA defines relatively broad permissions for the use of PHI that makes individual consent unnecessary in most cases. As such, establishing HIPAA-compliant practices come with significantly lower requirements than achieving GDPR-compliance. Furthermore, as the rules established by HIPAA cover only the aforementioned institutions, devices that are not intended to be used within the health care delivery context of one of the HIPAA-covered entities do not fall under these rules at all. Furthermore, some uncertainties may arise regarding the specific measures to be taken by the developers of sensors [52]. However, given that the importance of data privacy and security is often underestimated by manufacturers [53], familiarity and an early understanding of HIPAA rules is strongly advised for all manufacturers.

### 5.3. Privacy and Data Security for Smart Wearables

Smart wearables for cardiac monitoring that fulfil medical purposes are more than “just” lifestyle products, since they typically collect and process sensitive health care data. Given that, regulatory requirements often explicitly or implicitly include compliance with the corresponding privacy and data security rules, e.g., taking certain precautions for the data only to be accessible to the users of the smart wearables themselves or their health care providers. 

Article 110 of the European MDR, for example, explicitly requires the application of the GDPR and declares compliance with the GDPR’s rules mandatory for receiving a CE-mark. Compliance with the privacy and data security regulations becomes even more important if the wearable is sought to be used within a (public) health care system and reimbursed by health insurers. Because these institutions (as well as health care providers themselves) fall under prevailing domestic or regional data protection and security regulations, use of non-compliant devices is often prohibited and, at a minimum, would be expected to come with the cost of losing trust among patients and other parties in the health care system. Securing compliance with the respective privacy and data security rules of the target market, although potentially challenging, is of high importance for the successful market access of smart wearables in cardiac monitoring. 

## 6. Case Studies

This section presents a few brief case studies from the United States and European Union. They are not meant to be comprehensive, but rather representative, of the breadth of possibilities related to smart wearables for cardiac monitoring. All European case studies share the same market access criteria: a CE mark according to the EU’s MDR or, previously, MDD, respectively (see above for more detail). While the market access is harmonized for medical devices, including wearables, that are intended to be used for a medical purpose, the reimbursement conditions vary in each member state of the EU. In the following case studies, we will examine individual countries in more detail.

### 6.1. Case Study 1: Market Access in the USA

As noted above, all products that meet the definition of a medical device, as laid out in the Medical Device Amendments to the U.S. FD&C Act, are required to seek regulatory clearance (in the case of moderate-risk devices) or approval (in the case of high-risk devices) through the FDA. As in other countries, FDA clearance/approval is a necessary, but not sufficient, condition for reimbursement, which is determined by individual insurerssubject to complying with coverage for the ten “essential health benefits” (EHB) outlined in the 2010 Patient Protection and Affordable Care Act [54].

Until recently, coverage decisions in the United States were ad hoc and relatively limited. However, a 2019 final rule [55] from the U.S. Center for Medicare and Medicaid Services (CMS) established three new reimbursement codes for remote patient monitoring (RPM) for patients with chronic illnesses, these include, but are not limited to, cardiac conditions. The new codes facilitate reimbursement for the initial setup of RPM devices, associated patient education, the collection and interpretation of physiological data, and treatment management services for RPM.

As in several other areas of health care, the COVID-19 pandemic provided an additional boost to efforts around the deployment of digital products and applications. In particular, a 2020 update to the 2019 CMS rule further expanded coverage for RPM services [55]. The 2020 update also established an “add-on” code, allowing for the reimbursement of an additional 20 min of RPM services per month. Combined, these policies now allow U.S. providers to bill for up to 40 min of RPM services per Medicare patient monthly. Further, the CMS clarified that for the duration of the “national emergency” of the COVID-19 pandemic, RPM coverage would not be limited to chronic conditions, but could also include patients with acute conditions, such as COVID-19 itself [56]. While these policy updates broadly apply to RPM tools and services, many new areas of cardiac monitoring are already being impacted.

### 6.2. Case Study 2: Germany

In Germany, roughly 90% of the population (73 million individuals) is covered by the statutory health insurance system. Statutory health insurance is financed through the income-related contributions shared between employers and employees. Reimbursement decisions are taken by different bodies, depending on the type of product or service [57].

Until recently, there was no specific reimbursement scheme for digital health applications. However, in 2019, the Digital Health Care Act (DVG) established the “Fast-Track” process for medical devices of a lower risk category (I-IIa) that rely mainly on digital technologies that may include hardware, including wearables. These digital health applications, referred to as “DiGA” (based on their German acronym (“Digitale Gesundheitsanwendungen”)), are intended to be used by patients directly. They may be prescribed by physicians or psychotherapists in primary care [58].

**Figure 4:**
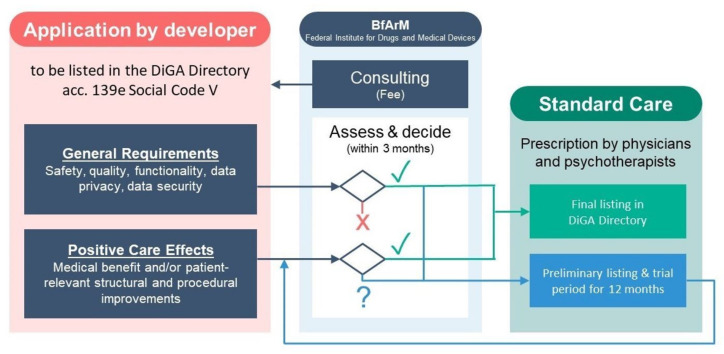
Fast track procedure for digital health applications in Germany.

Manufacturers apply to Germany’s Federal Institute for Drugs and Medical Devices (BfArM) in order to demonstrate compliance with pre-specified criteria on data privacy, security, quality, and safety. In addition, digital health applications must demonstrate evidence of “positive care effects”, either medical benefits (including traditional measures of morbidity and mortality) or so-called patient-related, structural or procedural improvements (including access to care, health literacy, adherence, patient safety, care coordination, and other patient-centered outcomes). Manufacturers may also choose to demonstrate positive care effects within a trial listing period of 12–24 months (Figure 4) [59]. 

### 6.3. Case Study 3: Belgium

In Belgium, most of the population is covered by the compulsory national health insurance system. The system is financed via social contributions, in relation to income. The National Institute for Health and Disability Insurance (NIHDI, Institut national d’assurance maladie-invalidité) manages compulsory health insurance, while privately organized non-profit sickness funds are in charge of the actual reimbursement of services. Reimbursed services (and possibly, necessary co-payments) are listed in the national fee schedule (la nomenclature des prestations de santé, INAMI). Reimbursement decisions are subject to negotiations between sickness funds and health care providers annually or every two years. Reimbursement decisions are based on therapeutic effects and budget impacts [60].

**Figure 5:**
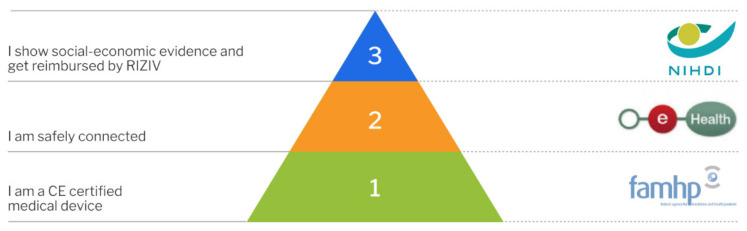
Validation Pyramid by mhealth Belgium, platform for CE-marked mobile health applications in Belgium [61]. Reprinted with permission from ref [61]. Copyright 2020 Agoria vzw/asbl and beMedTech vzw/asbl.

Since January 2019, mobile health applications must follow a specific validation scheme. The validation pyramid (Figure 5) consists of three tiers. The scheme applies to mobile health applications that collect, monitor, and share health-related information. For this, mobile health applications may use mobile devices, sensors, or health monitoring applications specifically designed to be used by the patient. The scheme may also include other medical devices designed to be used by the patient. Examples include MoveUp Coach, an app for treatment and rehabilitation for hip and knee arthroplasty, or the MySugr App, for diabetes management [61].

For reimbursement, mobile health applications, including those based on smart sensors, must pass through the requisite levels of validation (Figure 5). The first step consists of receiving a CE mark from the Federal Agency for Medicines and Health Products (FAMHP). In addition to the declaration as medical device, manufacturers need to demonstrate compliance with the GDPR (see above). In order to meet the requirements of the second level of validation, mobile health applications need to prove that they are safely connected. This includes the demonstration that the application meets all compulsory criteria, with regard to authentication, security, and the use of Belgian e-health services to guarantee interoperability, by means of standardized tests. Compliance is confirmed by an independent third party. The final level of the validation pyramid for mobile health applications requires the demonstration of “social-economic evidence”, which then leads to reimbursement by the National Institute for Health and Disability Insurance. A mobile health application may also choose not to pursue the third level of validation and, instead, look for other forms of financing/reimbursement, e.g., via hospitals, individual health care professionals, or marketing to patients directly [62].

## 7. Conclusions

As shown through the examples of the United States and two European countries, the requirements for market access (most importantly authorisation by the U.S. FDA or regulatory CE-marking via the conformity assessment procedure under the EU’s MDR), remuneration, and privacy/data security can differ significantly across countries and regions. Moreover, these requirements depend on the specificities of the product in question, its intended purpose, the technology used, the risks and benefits associated with its use, and the data it processes. 

Yet, the pathway to market access is characterised by many similar aspects and considerations at different stages. Although most of the requirements are formally considered and evaluated by Notified Bodies, authorities, or other competent authorities, or other competent bodies only after the product is fully developed and ready to be placed on the market, they should always be considered taken into account by developers and manufacturers early on during the new product development process for two key reasons. 

Firstly, early familiarity with market access regulations in the health care sector can help in assessing the potential (economic) success of a product. In many health care systems, meeting the requirements of health care providers, such as state authorities or health insurers, is the most important factor of success, as marketing a product only to self-paying individuals leaves the vast majority of patients that are not willing or able to pay for such products out-of-pocket behind. 

Secondly, many of the regulatory, legal, and market access requirements are much easier to comply with when considered early, at the time of product ideation, and during product development. Indeed, fulfilling data protection requirements, such as privacy by design, or regulatory demands, such as presenting comprehensive documentation of the software development process, can present a resource-intensive challenge to manufacturers. Dealing with these requirements only after the product has been developed may lead to a high risk of insufficient information in audit-relevant documentation and prohibitively high costs.

The current status of the regulatory and legal aspects for smart wearables is characterised by a changing landscape: in the near future, the market for wearables in cardiac monitoring will expand further, as it appears that wearables will also play a significant role as a means to collect data, upon which clinical predictive models can be based and trained [63,64]. This mostly untapped market creates the potential for both researchers and the industry to use wearables as part of a feedback loop to further improve the development of such tools. This, in turn, will improve cardiac monitoring solutions for clinical treatment. 

Many of the same features apply to the market for medical device software, including SaMD, as well as digital solutions for teleconsultation and remote patient monitoring in general. The rise of new technologies will, in some settings, lead to the creation and updating of policies to allow for their use in formal health care delivery systems. On the regulatory side, in Europe, the MDR contains new rules concerning software with an intended medical purpose, increasing market access barriers while allowing a means to address software specificities that were mostly neglected or, in the worst cases, led to extensive legal disputes. With the changing regulatory landscape under the MDR, new approaches to collaboration between manufacturers and clinicians might help to meet regulatory requirements in a timely manner and develop new health care products that provide added value for patients. 

To accelerate innovation and medical product development in the U.S. context, the FDA has released a framework to implement its real-world evidence (RWE) program to support the use of real-world data (RWD) and, thereby, RWE to support the FDA’s regulatory decision-making [65]. The RWE generated by RWD can be used by manufacturers to support clinical trial designs for innovative treatment approaches, such as the use of smart wearables in the field of cardiovascular diseases. 

In Germany, the novel DiGA Fast-Track (see case study above) takes a similar approach, turning away from strict up-front RCT requirements for the reimbursement of specific software medical devices, in favour of a more dynamic HTA approach. This is expected to allow manufacturers to provide evidence in a 12–24 month trial period, also based (at least in part) on RWD, when such an approach is more feasible or appropriate.

The reality of many novel medical technologies is that health care systems and their regulations were established before the advent of many of today’s most innovative medical products. This is specifically true in considering the rise of digital technologies and corresponding smart wearables, which can be used in health care and are easy to include in patients’ daily lives. As such, the evolution of, and changes to, legislation and regulation will need to be considered on an ongoing basis. We are optimistic that the ongoing efforts of legislators to drive policy innovations that include digitally driven health care technologies (such as smart wearables) in health care systems and in patients’ lives will continue.

## Figures and Tables

**Table 1:** Examples of consumer, clinical, and research-grade wearable devices.

Manufacturer	Product Name	Description	Measurements	Market Access	References
Apple Inc.	Apple Watch Series 4–6 ECG-App	Software paired with specific device type (Apple Watch Series 4–6)	ECG, Oxygen Saturation, Accel	FDA Cleared/CE Marked	[5]
AliveCor	Kardia Band	Wristband	ECG	FDA Cleared	[6]
Ava Science, Inc.	Ava Wristband	Wristband	Accel, PPG HR, temperature sensors	FDA Approved	[7]
Fitbit	Charge/Sense	Software paired with specific device type (Fitbit Charge 2–3, Sense)	Accel, PPG HR	FDA Cleared/CE Marked	[8]
Omron	Heart Guide	Watch	Accel, PPG HR, oscillometric bloodpressure	FDA Approved	[9]
Withings	ScanWatch	Watch	ECG, Oxygen Saturation, HR, Accel	FDA Cleared/CE Marked	[10]
iRhythm	Ziopatch	Chest patch	ECG	FDA Cleared	[11]
Motiv	Motiv Ring	Ring	Accel, PPG HR	Consumer grade	[12]
Preventice	Bodyguardian Heart	Chest patch	Accel, ECG	FDA Cleared	[13]

ECG = Electrocardiography, Accel = Accelerometer, PPG = Photoplethysmography, HR = Heart Rate, FDA = Food and Drug Administration, CE = Conformitè Europëenne (EN, European Conformity). Table adapted and updated with recent data from [14].

**Table 2:** Comparison of EU-MDR and FD&C Act definitions of medical devices.

Medical Device Definition acc. EU-MDR 2017/745, Art. 2(1)	Medical Device Definition under Section 201(h) of the FD&C Act
“Any instrument, apparatus, appliance, software, implant, reagent, material, or other article’ intended by the manufacturer to be used, alone or in combination, for human beings for one or more of the following specific medical purposes: - Diagnosis, prevention, monitoring, prediction, prognosis, treatment, or alleviation of disease - Diagnosis, monitoring, treatment, alleviation of, or compensation for, an injury or disability - Investigation, replacement, or modification of the anatomy or of a physiological or pathological process or state - Providing information by means of in vitro examination of specimens derived from the human body, including organ, blood, and tissue donations, and which does not achieve its principal intended action by pharmacological, immunological, or metabolic means, in or on the human body, but which may be assisted in its function by such means. […]” (MDR, Art. 2(1)) [20]	“An instrument, apparatus, implement, machine […] which is intended for use in the diagnosis of disease or other conditions, or in the cure, mitigation, treatment, or prevention of disease […] which does not achieve its primary intended purposes through chemical action within or on the body of man […] and which is not dependent upon being metabolized for the achievement of its primary intended purposes […]” (201(h) of the FD&C Act) [21]

## Data Availability

Not applicable.
